# Evaluating TikTok and YouTube as patient-education resources on kidney transplantation: a comparative analysis

**DOI:** 10.3389/fpubh.2026.1764270

**Published:** 2026-03-13

**Authors:** Runmin Ding, Junyi Zhou, Huan Tang, Lu Shi, Zijie Wang, Min Gu, Zhonglei Deng, Zeping Gui, Zhiwang Tang

**Affiliations:** 1 Department of Urology, Jiangsu Key Laboratory of Urological Disease Prevention and Treatment, The Second Affiliated Hospital of Nanjing Medical University, Nanjing Medical University, Nanjing, China; 2 The Second Clinical Medical College, Nanjing Medical University, Nanjing, China; 3 Department of Urology, The Affiliated Huaian No.1 People's Hospital of Nanjing Medical University, Huaian, China

**Keywords:** internet, kidney transplantation, patient education, quality, TikTok, YouTube

## Abstract

**Objective:**

This study evaluated and compared the quality and educational value of kidney transplantation-related videos on TikTok and YouTube.

**Methods:**

A structured search identified 151 eligible videos. Each video was assessed using DISCERN, PEMAT-A/V (understandability and actionability), and the Global Quality Scale (GQS). Content completeness was examined across six key educational domains. Correlation analyses were conducted to determine associations between video characteristics and quality metrics.

**Results:**

Physicians were the primary uploaders on both platforms (YouTube 55.7%, TikTok 46.0%). Overall quality was low: 69.4% of TikTok and 62.8% of YouTube videos were rated “poor” or “very poor.” Only 2.3% of YouTube videos achieved an “excellent” rating, and none on TikTok. TikTok showed much higher engagement (mean 13,639 likes and 2,664 comments per video) than YouTube (1,480 likes and 51 comments), yet engagement and duration were not correlated with quality on TikTok (*p* > 0.5). In contrast, YouTube video duration was positively associated with DISCERN and GQS scores (*r* = 0.478–0.584, *p* < 0.001). Content completeness was limited on both platforms, particularly for evaluation and long-term outcomes, and TikTok demonstrated significantly lower scores across all domains. Significant between-platform differences were observed in DISCERN (*p* < 0.001), GQS (*p* = 0.001), PEMAT understandability (*p* = 0.002), and content completeness (*p* < 0.01).

**Conclusion:**

TikTok and YouTube provide suboptimal educational content on kidney transplantation, with TikTok performing notably worse. Greater involvement of medical institutions and improved platform mechanisms to elevate high-quality, evidence-based content are urgently needed to enhance online patient education.

## 1 Introduction

Kidney transplantation constitutes the majority of organ transplants performed globally each year, representing 64.46% of approximately 170,000 procedures, according to 2023 statistics from World Health Organization (WHO) and Global Observatory on Donation and Transplantation (GODT) (1). Kidney transplantation is widely regarded as the most effective treatment for end-stage renal disease (ESRD), as it offers substantial benefits in terms of patient survival, quality of life, and long-term cost-effectiveness when compared with maintenance dialysis (2). In recent decades, advances in immunosuppressive therapy and rejection-risk stratification have markedly improved short-term outcomes, with one-year post-transplant survival now exceeding 90%. However, long-term graft survival remains a persistent clinical challenge. Among recipients of deceased-donor kidneys, the 10-year graft survival rate is only about 50%, and even in cases of living-donor transplantation, long-term survival does not exceed approximately 70% (3, 4).

Successful transplantation requires lifelong adherence to complex immunosuppressive regimens, lifestyle adjustments, and regular follow-up care (5). Numerous studies have identified limited health literacy as a major barrier to achieving optimal transplant outcomes, as low adherence is associated with an increased risk of graft loss, hospital readmission, and mortality (6–8).

In recent years, patients have increasingly turned to digital platforms, especially social media, for more accessible health information (9). YouTube and TikTok have gained considerable prominence owing to their video-based formats, user-generated content, and broad global reach. YouTube has over 2 billion active users worldwide, while TikTok, launched more recently, surpassed 1.5 billion users by 2023, the majority of whom are under the age of 35 (10, 11). While these platforms provide opportunities to broaden access to health education and engage younger audiences, the absence of expert review mechanisms has prompted serious concerns about misinformation, content oversimplification, and inconsistency in information quality (12).

Previous researches evaluating transplantation-related videos on YouTube has shown that the overall educational quality is often suboptimal, with frequent issues such as incomplete coverage of key clinical information and inconsistent medical accuracy (13, 14). In contrast, studies examining TikTok's role in health education remain scarce, despite the platform's rapid growth and substantial popularity among adolescents and young adults (15–17). Notably, no prior investigation has systematically compared kidney transplantation-related content across these two platforms. Comparable concerns have also been reported across other medical specialties. In a recent systematic evaluation of cardiac rehabilitation-related content on YouTube, Tezcan et al. (18) found that although many videos achieved high engagement, their educational quality and reliability were often inadequate. These findings suggest that the discrepancy between popularity metrics and informational value may reflect a broader pattern across medical domains rather than being limited to a single specialty.

In light of the urgent need for accurate and comprehensive transplant education, especially among younger and digitally engaged patient populations, a systematic evaluation of the quality and reliability of online video-based information is warranted. This study aims to assess and contrast kidney transplantation-related videos on YouTube and TikTok, employing established evaluation tools to measure quality, reliability, and content completeness. By identifying existing gaps and platform-specific limitations, this research seeks to inform targeted strategies to improve digital transplant education and enhance evidence-based communication strategies in the digital health domain.

## 2 Materials and methods

### 2.1 Search strategy and data extraction

A structured search was conducted on TikTok (Douyin in China) and YouTube to identify videos related to kidney transplantation. The search was performed on October 30th, 2025 using the Chinese keyword “肾移植” on TikTok and the English keyword “kidney transplantation” on YouTube. These terms were selected based on standard clinical terminology and commonly used descriptors in transplantation-related literature to maximize retrieval of relevant content. To minimize personalization bias and ensure reproducibility, browsing history, cache, and cookies were cleared prior to performing the searches. Videos were sorted according to each platform's default relevance-based ranking algorithm, and the first 100 videos from each platform were screened for eligibility.

**Figure 1:**
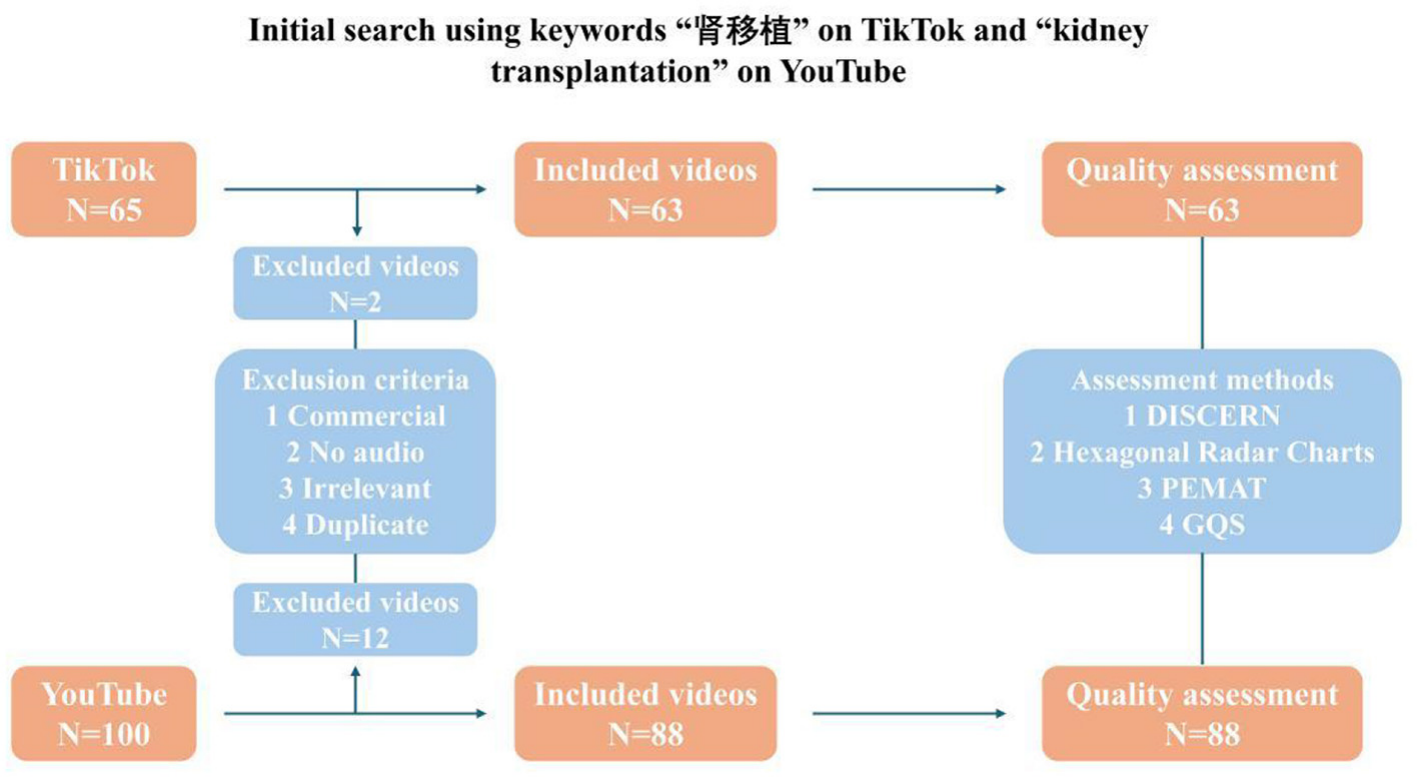
Flowchart of the selection of videos included in the analysis.

Two independent reviewers screened all retrieved videos according to predefined inclusion and exclusion criteria. Inclusion criteria were: (1) the video must contain educational content primarily related to kidney transplantation, including any aspect such as surgical process, donor information, post-operative care, or medication; (2) the video must be in Mandarin (TikTok) or English (YouTube); and (3) the video must contain either audio narration or subtitles. Videos were excluded if they were (1) commercial advertisements, (2) duplicated content, (3) off-topic or irrelevant, or (4) primarily entertainment-focused without any educational value. Disagreements between reviewers were resolved through discussion until consensus was reached. After screening, 63 videos from TikTok and 88 videos from YouTube met the inclusion criteria and were included for analysis. For each video, metadata including URL, upload date, video duration, view count, number of likes, comments, and type of uploader (e.g., physician, non-profit organization, individual user, commercial entity) were recorded using Microsoft Excel for subsequent analysis (Figure 1).

### 2.2 Assessment procedure

All selected videos were evaluated independently by two trained raters (DRM and ZJY) based on both content completeness and quality of health information. Any discrepancies in the ratings were resolved through discussion with a third rater (TH) until consensus was reached.

To assess content completeness, each video was evaluated across six key informational domains: (1) basic understanding of kidney transplantation (e.g., procedure overview, candidacy), (2) donor-related information (e.g., living or deceased donors, matching criteria), (3) surgical process and hospitalization, (4) post-transplant care (e.g., hygiene, lifestyle modifications), (5) complications and risk of rejection, and (6) medication and immunosuppression (basic introduction). Each domain was scored on a five-point scale: 0 for no content, 0.5 for minimal content, 1 for moderate content, 1.5 for substantial content, and 2 for comprehensive coverage. These scores were later visualized using a hexagonal radar chart to compare content distribution across platforms.

Video quality was evaluated using three standardized tools. The DISCERN instrument was applied to assess the reliability and quality of treatment-related information, with 16 questions rated on a scale from 1 (poor) to 5 (excellent), resulting in a total score range of 16 to 80. Scores were categorized into five levels: very poor (< 27), poor (27–38), fair (38–50), good (51–62), and excellent (63–80). The Patient Education Materials Assessment Tool for Audiovisual Materials (PEMAT-A/V) was used to measure understandability (13 items) and actionability (4 items). Scores were calculated as a percentage of items rated “agree” and were considered satisfactory when ≥70%. The Global Quality Scale (GQS), a 5-point Likert scale, was employed to rate the overall quality, usefulness, and flow of information in each video, with scores of 1–2 indicating low quality, 3 moderate quality, and 4–5 high quality.

### 2.3 Inter-rater reliability

All videos were assessed independently by three trained raters using the DISCERN, PEMAT, and GQS instruments. To assess the consistency among raters, inter-rater reliability was calculated using intraclass correlation coefficients (ICC) based on a two-way random-effects model with absolute agreement. As the final analysis was based on the mean scores of the three raters, average-measure ICC values were reported. ICC values were interpreted as follows: < 0.50, poor; 0.50–0.75, moderate; 0.75–0.90, good; and >0.90, excellent agreement.

### 2.4 Statistical analysis

All statistical analyses were performed using IBM SPSS Statistics version 26.0 (IBM Corp., Armonk, NY, USA). Continuous variables were expressed as means ± standard deviation (SD), while categorical variables were presented as frequencies and percentages. The independent sample *t*-test was used for comparing continuous variables with normal distribution, while the Kruskal–Wallis test was used for non-parametric data.

Spearman's rank correlation coefficient was applied to examine associations between video characteristics (such as duration, number of likes, and comments) and video quality metrics (DISCERN, PEMAT, and GQS scores). A *p*-value of less than 0.05 was considered statistically significant in all analyses.

## 3 Results

### 3.1 Video characteristics

In total, 151 kidney transplantation-related videos sourced from YouTube and TikTok were analyzed (Table 1). The average video duration on YouTube was significantly longer than that on TikTok (438.30 ± 852.03 seconds vs. 152.02 ± 138.65 seconds, *p* = 0.012). Similarly, YouTube videos had been available online longer (1420.84 ± 1317.61 days) compared to TikTok videos (383.17 ± 343.91 days), with a statistically significant difference (*p* < 0.001). Regarding popularity, due to platform restrictions, data on total views and views per day were unavailable for TikTok. YouTube videos, however, had an average of 171,973.69 ± 926,781.68 views, with a mean of 126.80 ± 321.73 daily views. TikTok videos showed higher average like counts (13,639.29 ± 26,233.91) than YouTube (1,480.30 ± 9,509.02), though the difference was not statistically significant (*p* = 0.575). Regarding uploader characteristics, YouTube demonstrated a more diverse distribution of uploader types, with physicians constituting the majority, followed by non-profit organizations and individual users. In contrast, TikTok videos were predominantly uploaded by physicians.

**Table 1:** General features of included videos.

**Source of upload**	**YouTube**		**TikTok**		***P*-value**
	**N**	**%**	**N**	**%**	
Physician	48	55.68	29	46.03	–
Normal user	1	1.14	32	50.79	–
Non–profit organization	39	44.31	2	3.17	–
**Video features**	**Mean** ±**Std. Deviation**	**Min–Max**	**Mean** ±**Std. Deviation**	**Min–Max**	–
Duration(s)	438.30 ± 852.03	25.00–6,020.00	152.02 ± 138.65	15.00–524.00	0.012
Number of days online	1,420.84 ± 1,317.61	1.00–5,961.00	383.17 ± 343.91	5.00–1,092.00	< 0.001
Number of views	171,973.69 ± 926,781.68	262.00–8,406,772.00	–	–	–
Number of views/day	126.80 ± 321.73	0.001–2,402.62	–	–	–
Number of likes	1,480.30 ± 9,509.02	0.00–88,000.00	13,639.29 ± 26,233.91	6.00–122,200.00	0.575
Number of likes/day	1.68 ± 4.70	0.00–39.00	205.34 ± 359.71	0.01–1,545.00	< 0.001
Number of comments	50.93 ± 127.07	0.00–827.00	2,664.12 ± 5,930.34	0.00–26,800.00	0.574
Number of comments/day	0.21 ± 1.14	0.00–13.00	44.57 ± 95.83	0.00–434.00	< 0.001
Number of collects	–	–	830.40 ± 1,664.44	0.00–7,200.00	–
Number of collects/day	–	–	15.34 ± 33.10	0.00–148.00	–
DISCERN reliability	20.76 ± 6.24	10.33–34.00	20.35 ± 5.63	8.33–30.67	< 0.001
DISCERN treatment	11.78 ± 4.71	5.00–27.67	11.23 ± 3.86	5.00–20.67	< 0.001
DISCERN quality	2.35 ± 0.97	1.00–4.67	2.21 ± 0.99	1.00–4.00	< 0.001
DISCERN	36.90 ± 12.43	19.67–69.00	33.79 ± 9.07	18.00–54.00	< 0.001
PEMAT understandability total points	5.79 ± 3.56	1.00–13.33	4.60 ± 3.01	0.00–11.00	0.003
PEMAT understandability total possible points	14.00 ± 0.00	14.00–14.00	14.28 ± 0.81	14.00–16.00	–
PEMAT understandability score (%)	0.41 ± 0.25	0.07–0.95	0.32 ± 0.20	0.00–0.79	0.002
PEMAT actionability total points	1.02 ± 0.89	0.00–2.67	0.70 ± 0.69	0.00–2.33	0.115
PEMAT actionability total possible points	4.00 ± 0.00	4.00–4.00	4.00 ± 0.00	4.00–4.00	–
PEMAT actionability score (%)	0.25 ± 0.22	0.00–0.67	0.18 ± 0.17	0.00–0.58	0.120
GQS	2.47 ± 1.08	1.00–5.00	2.05 ± 0.82	1.00–4.00	0.001

### 3.2 Video quality and content

#### Video quality evaluation

**Figure 2:**
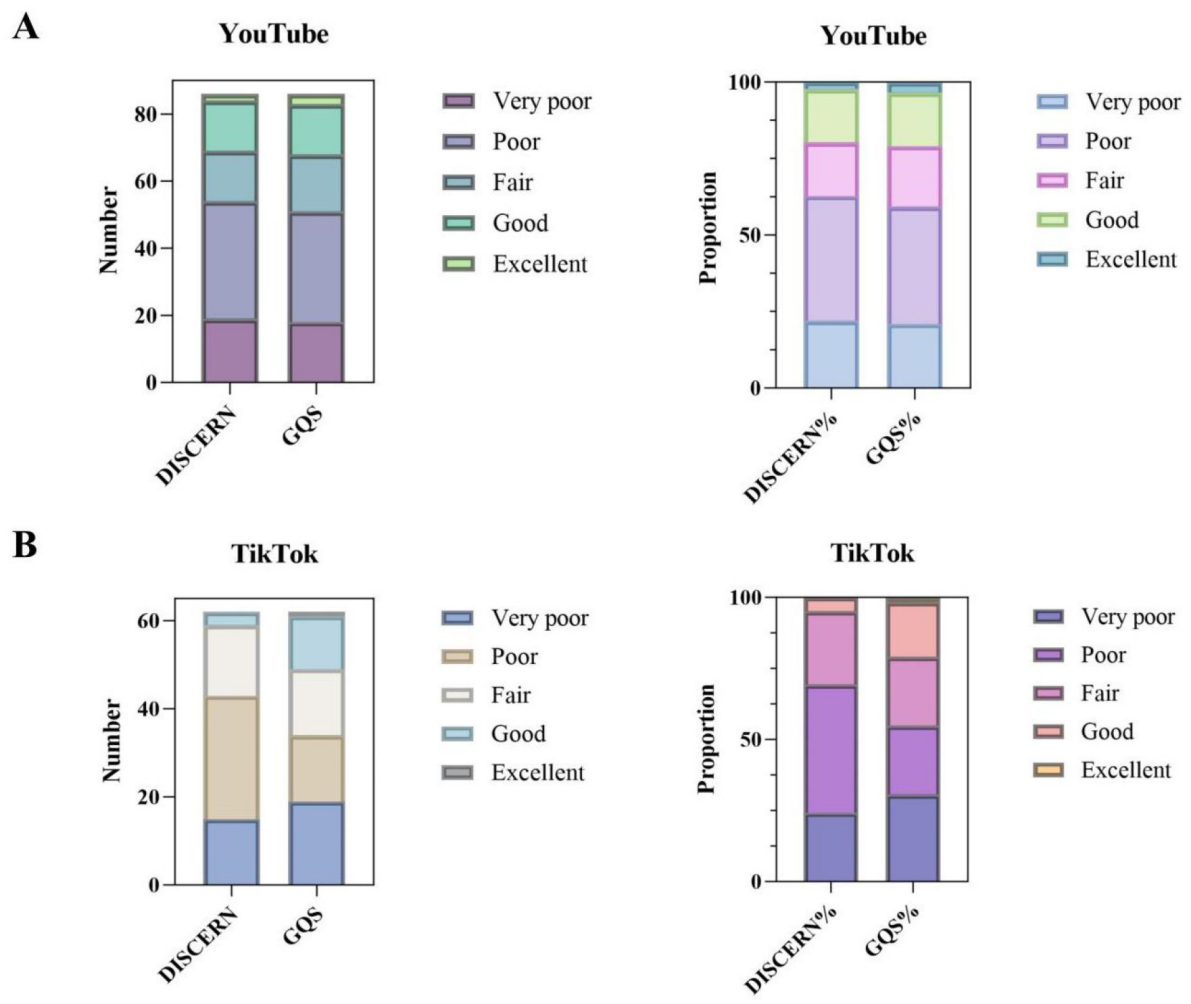
Number and proportion of the 5 levels of DISCERN and the Global Quality Score (GQS). **(A)** YouTube. **(B)** TikTok.

The DISCERN tool was used to assess and classify video quality into five categories (Table 2, Figure 2). On YouTube, 22.1% of videos were rated as “very poor,” 40.7% as “poor,” 17.4% as “fair,” 17.4% as “good,” and 2.3% as “excellent.” On TikTok, the proportion of “very poor” (24.2%) and “poor” (45.2%) quality videos was higher, while only 4.8% were rated as “good,” and none were rated as “excellent.” YouTube videos rated as “good” or “excellent” had considerably longer durations (990.93 ± 1498.47 s and 894.00 ± 1094.60 s, respectively) than those rated as “poor” or “very poor.” However, the differences in video length across DISCERN categories were not statistically significant (*p* > 0.3). For TikTok, no video was rated as “excellent,” and no clear trend was observed between video duration and quality rating.

**Table 2:** Distribution of DISCERN classification according to the video features.

**Variable**	**Platform**	**Statistic**	**Very poor**	**Poor**	**Fair**	**Good**	**Excellent**	***P*-value**
Number of videos	YouTube		19 (22.1%)	35 (40.7%)	15 (17.4%)	15 (17.4%)	2 (2.3%)	N/A
Number of videos	TikTok		15 (24.2%)	28 (45.2%)	16 (25.8%)	3 (4.8%)	0	N/A
Duration(s)	YouTube	Mean ± SD	143.42 ± 95.87	340.77 ± 754.78	426.00 ± 427.11	990.93 ± 1,498.47	894.00 ± 1,094.60	0.321
		Median ± IQR	124.00 ± 76.00	141.00 ± 160.00	323.00 ± 332.50	396.00 ± 768.00	894.00 ± 774.00	
	TikTok	Mean ± SD	268.50 ± 348.54	138.70 ± 88.20	136.29 ± 62.06	333.33 ± 136.57	N/A	0.617
		Median ± IQR	122.0 ± 113.75	118.0 ± 106.00	129.00 ± 63.00	257.0 ± 119.50	N/A	
Number of views	YouTube	Mean ± SD	22,876.16 ± 34,058.25	305,531.77 ± 1,425,395.25	70,789.67 ± 160,209.14	161,586.47 ± 408,024.42	87,918.00 ± 44,745.72	0.423
		Median ± IQR	5,110.00 ± 28,023.50	8,802.0 ± 39,156.00	14,138.00 ± 28,382.00	9,284.00 ± 114,178.50	87,918.00 ± 31,640.00	
	TikTok	Mean ± SD	N/A	N/A	N/A	N/A	N/A	N/A
		Median ± IQR	N/A	N/A	N/A	N/A	N/A	
Number of views/day	YouTube	Mean ± SD	79.29 ± 289.75	125.45 ± 417.45	131.37 ± 440.85	197.44 ± 372.25	37.90 ± 12.10	0.230
		Median ± IQR	5.96 ± 24.02	13.75 ± 31.92	8.08 ± 14.04	12.83 ± 147.21	37.90 ± 8.55	
	TikTok	Mean ± SD	N/A	N/A	N/A	N/A	N/A	N/A
		Median ± IQR	N/A	N/A	N/A	N/A	N/A	
Number of likes	YouTube	Mean ± SD	166.84 ± 291.56	2,877.11 ± 14,837.14	354.60 ± 665.76	1,186.27 ± 2,210.50	162.00 ± 229.10	0.743
		Median ± IQR	36.00 ± 75.50	76.00 ± 366.00	111.00 ± 148.00	59.00 ± 1,374.50	162.00 ± 162.00	
	TikTok	Mean ± SD	1,494.53 ± 3,326.86	5,234.61 ± 19,026.28	2,197.06 ± 2,407.01	1,652.67 ± 1,520.79	N/A	0.244
		Median ± IQR	339.00 ± 1,217.50	1,795.00 ± 1,964.75	1,660.50 ± 1,268.00	1,792.00 ± 1,516.00	N/A	
Number of likes/day	YouTube	Mean ± SD	2.13 ± 8.93	1.15 ± 4.32	1.25 ± 4.55	2.97 ± 6.63	0.08 ± 0.12	0.186
		Median ± IQR	0.03 ± 0.12	0.11 ± 0.39	0.06 ± 0.11	0.18 ± 2.19	0.08 ± 0.08	
	TikTok	Mean ± SD	79.26 ± 184.75	137.87 ± 401.98	23.74 ± 22.29	2.70 ± 3.18	N/A	0.101
		Median ± IQR	17.43 ± 52.05	8.26 ± 19.06	15.22 ± 20.46	1.58 ± 3.03	N/A	
Number of comments	YouTube	Mean ± SD	61.68 ± 189.86	54.57 ± 123.16	35.33 ± 79.83	49.40 ± 88.33	13.50 ± 19.09	0.986
		Median ± IQR	3.00 ± 11.5	6.00 ± 34.5	7.00 ± 13.50	2.00 ± 65.00	13.50 ± 13.50	
	TikTok	Mean ± SD	511.80 ± 1,354.16	998.43 ± 3,686.94	318.19 ± 512.17	610.33 ± 969.84	N/A	0.969
		Median ± IQR	189.00 ± 226.5	146.50 ± 330.25	168.00 ± 180.00	96.00 ± 861.50	N/A	
Number of comments/day	YouTube	Mean ± SD	0.03 ± 0.10	0.08 ± 0.22	0.01 ± 0.02	0.96 ± 3.34	0.01 ± 0.01	0.807
		Median ± IQR	0.00 ± 0.01	0.01 ± 0.06	0.00 ± 0.02	0.00 ± 0.09	0.01 ± 0.01	
	TikTok	Mean ± SD	26.39 ± 76.15	17.47 ± 43.82	2.60 ± 2.69	0.41 ± 0.43	N/A	0.018
		Median ± IQR	3.67 ± 4.70	1.23 ± 1.95	1.45 ± 2.01	0.34 ± 0.43	N/A	
Number of collects	YouTube	Mean ± SD	N/A	N/A	N/A	N/A	N/A	N/A
		Median ± IQR	N/A	N/A	N/A	N/A	N/A	
	TikTok	Mean ± SD	136.00 ± 312.74	426.00 ± 1,027.28	452.06 ± 436.23	353.33 ± 391.87	N/A	0.022
		Median ± IQR	27.00 ± 62.50	173.00 ± 368.00	384.00 ± 413.25	255.00 ± 382.50	N/A	
Number of collects/day	YouTube	Mean ± SD	N/A	N/A	N/A	N/A	N/A	N/A
		Median ± IQR	N/A	N/A	N/A	N/A	N/A	
	TikTok	Mean ± SD	8.51 ± 17.63	17.44 ± 66.93	4.75 ± 4.12	0.98 ± 1.53	N/A	0.180
		Median ± IQR	1.15 ± 6.59	0.98 ± 2.91	3.76 ± 5.75	0.13 ± 1.34	N/A	
PEMAT understandability score (%)	YouTube	Mean ± SD	16.67 ± 5.83	38.10 ± 19.45	56.19 ± 20.54	0.63 ± 0.27	55.95 ± 48.83	< 0.001
		Median ± IQR	16.67 ± 4.76	33.33 ± 20.24	50.00 ± 30.95	71.43 ± 39.29	55.95 ± 34.52	
	TikTok	Mean ± SD	22.54 ± 10.91	31.68 ± 13.71	42.06 ± 18.33	0.54 ± 0.25	N/A	0.004
		Median ± IQR	23.81 ± 15.78	31.25 ± 20.24	43.30 ± 17.71	54.76 ± 25.00	N/A	
PEMAT actionability score (%)	YouTube	Mean ± SD	10.96 ± 13.90	17.86 ± 18.65	40.00 ± 15.49	0.45 ± 0.21	41.67 ± 35.36	< 0.001
		Median ± IQR	8.33 ± 16.67	8.33 ± 33.33	41.67 ± 25.00	50.00 ± 20.83	41.67 ± 25.00	
	TikTok	Mean ± SD	22.96 ± 20.34	26.19 ± 20.78	45.83 ± 24.30	0.70 ± 0.06	N/A	0.002
		Median ± IQR	22.22 ± 27.78	27.78 ± 33.33	50.00 ± 33.34	66.67 ± 5.55	N/A	
GQS	YouTube	Mean ± SD	1.38 ± 0.29	2.27 ± 0.76	3.16 ± 0.73	3.56 ± 1.04	3.17 ± 2.12	< 0.001
		Median ± IQR	1.33 ± 0.50	2.0 ± 1.0	3.00 ± 1.00	4.0 ± 1.33	3.17 ± 1.50	
	TikTok	Mean ± SD	1.44 ± 0.63	2.33 ± 1.02	3.25 ± 0.99	4.11 ± 0.19	N/A	< 0.001

#### Correlation analysis

As shown in Table 3, on YouTube, DISCERN and GQS scores demonstrated a strong positive correlation (*r* = 0.759, *p* < 0.001) and were both positively associated with video duration (*r* = 0.478 and *r* = 0.584, respectively; both *p* < 0.001). On TikTok, the correlation between DISCERN and GQS scores was also statistically significant (*r* = 0.659, *p* < 0.001), but video duration was not significantly correlated with either metric (*p* > 0.5). These findings suggest that longer YouTube videos tended to provide higher-quality and more reliable content, whereas TikTok video quality did not show a similar association with duration.

**Table 3:** Correlation analyses for DISCERN score and GQS score.

**Variable**	**YouTube**				**TikTok**			
	**DISCERN**		**GQS**		**DISCERN**		**GQS**	
	***r***	***P*** **-value**	***r***	***P*** **-value**	***r***	***P*** **-value**	***r***	***P*** **-value**
DISCERN	–	–	0.759	**<0.001**	–	–	0.659	< 0.001
GQS	0.759	**<0.001**	–	–	0.659	**<0.001**	–	–
Duration(s)	0.478	**<0.001**	0.584	**<0.001**	0.089	0.524	**0.016**	0.910
Number of likes	0.101	0.357	0.054	0.623	0.173	0.180	−0.072	0.580
Likes/day	0.144	0.187	**-0.02**	0.854	−0.057	0.661	−0.094	0.466
Number of comments	**-0.064**	0.559	−0.166	0.126	−0.056	0.665	−0.130	0.315
Comments/day	**-0.031**	0.780	−0.184	0.090	−0.323	**0.011**	−0.232	0.070
PEMAT understandability score (%)	0.689	**<0.001**	0.885	**<0.001**	0.410	**0.002**	0.417	**0.002**
PEMAT actionability score (%)	0.623	**<0.001**	0.810	**<0.001**	0.408	**0.002**	0.310	**0.022**

Bold values indicate statistically significant correlations (*p* < 0.05).

#### Inter-rater reliability

For YouTube videos, the average-measure ICC demonstrated good agreement across evaluation tools, with ICC values of 0.72 for DISCERN, 0.80 for PEMAT, and 0.83 for GQS. For TikTok videos, agreement was moderate for DISCERN (ICC = 0.68) and PEMAT (ICC = 0.63), while excellent agreement was observed for GQS (ICC = 0.91). Overall, these findings indicate acceptable to strong inter-rater reliability across both platforms.

#### Content completeness

**Figure 3:**
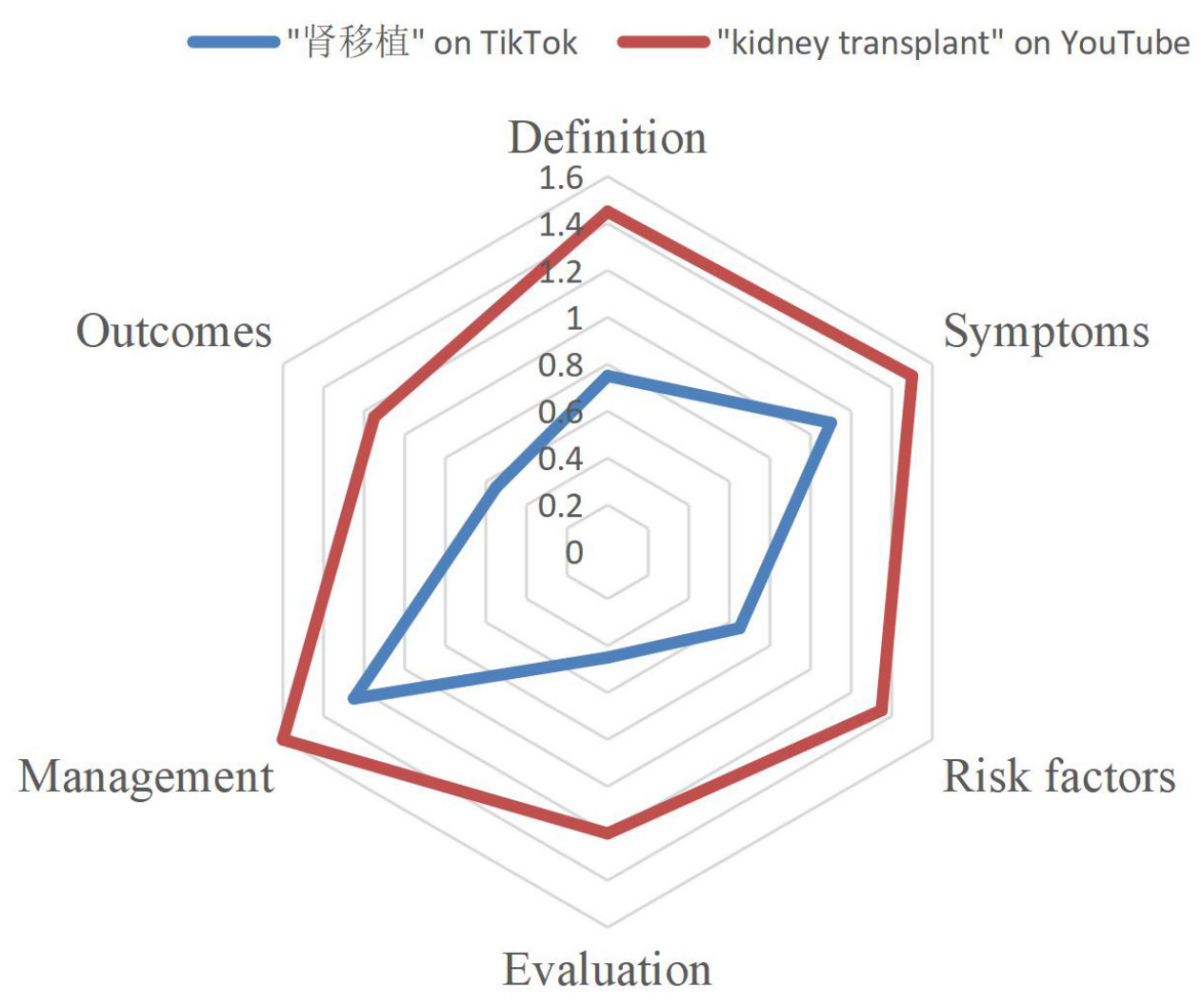
Completeness of video content.

Video content was evaluated across six key content domains: definition, symptoms, risk factors, evaluation, management, and outcomes. YouTube videos consistently scored higher than TikTok videos across all six dimensions (Table 4, Figure 3). For instance, the mean scores for “definition” and “management” were 1.45 and 1.60 on YouTube vs. 0.75 and 1.25 on TikTok, respectively. The most frequently addressed topics on both platforms were “symptoms” and “management,” whereas “evaluation” and “outcomes” received notably less attention, particularly on TikTok, where the mean scores were 0.45 and 0.55, respectively. The radar chart demonstrated that TikTok videos exhibited limited depth of content across all domains, with no topic achieving a mean score above 1.10. In contrast, YouTube content showed more comprehensive and balanced coverage, especially in critical areas such as medication management and donor evaluation.

**Table 4:** Completeness of video content.

**Item**	**Definition**	**Symptoms**	**Risk factors**	**Evaluation**	**Management**	**Outcomes**
“肾移植” on TikTok	0.75	1.10	0.65	0.45	1.25	0.55
“kidney transplant” on YouTube	1.45	1.50	1.35	1.20	1.60	1.15

## 4 Discussion

To our knowledge, this is the first study to systematically assess and compare the educational quality of kidney transplantation-related videos on TikTok and YouTube. Although major social media platforms are often considered potential tools for patient education, our findings demonstrate that both TikTok and YouTube fall short in providing high-quality, comprehensive health information, with TikTok performing significantly worse across nearly all evaluated domains (19).

The overall quality of the content, as measured by the DISCERN instrument, remained generally low on both platforms. In our sample, 68% of TikTok videos were rated as “poor” or “very poor,” and none met the criteria for “excellent.” YouTube showed relatively better performance, with 19.7% of videos achieving “good” or “excellent” ratings, although most still lacked reliability, balance, and informational depth. These findings are in line with previous TikTok-based evaluations, such as Sun et al. (16), who reported consistently low DISCERN and GQS scores in gallstone-related videos, highlighting the platform's limited capacity to deliver reliable medical information.

A critical concern highlighted by our analysis is the insufficient presence of professionally sourced content. While 80% of TikTok videos were uploaded by physicians, the majority received low scores in quality and completeness. This paradox may be explained by platform-specific features, particularly TikTok's emphasis on short-form video formats and engagement-focused algorithms, which prioritize viewer retention at the expense of educational depth. Comparable trends have been observed in other areas of medical communication. Similar trends have been documented in other medical domains. Liu et al. (20) reported that although 94.9% of chronic renal failure–related TikTok videos were created by healthcare professionals, most still fell within the low-to-moderate quality range on DISCERN and GQS. A similar pattern was reported by Wang et al. (21), who found that TikTok videos on Hashimoto's thyroiditis, although predominantly produced by verified physicians, performed poorly across multiple evaluation tools, including PEMAT, mDISCERN, GQS, and JAMA criteria. These findings reinforce the notion that platform-specific constraints may hinder the educational quality of health content, even when it is created by qualified medical professionals.

Regarding content completeness, both platforms were disproportionately focused on introductory topics. Although 82% of videos addressed general symptoms or lifestyle advice, fewer than 20% discussed essential topics such as diagnostic evaluation, immunosuppressive protocols, or long-term complications. This observation aligns with findings from Sturm et al., who reported that TikTok videos related to pediatric kidney disease and transplantation rarely included treatment- or management-related information, with most content focusing on introductory or non-clinical topics despite substantial viewer engagement (22). Such imbalances may contribute to the misconception that kidney transplantation is a routine, low-maintenance procedure, which could, in turn, undermine patient adherence and long-term postoperative vigilance.

Another key finding of this study is the disconnect between user engagement and video quality. Although TikTok videos received significantly higher average like counts than those on YouTube, they consistently scored lower on DISCERN, GQS, and content-completeness measures. This pattern is consistent with prior researches. Liu et al. (23) found no strong relationship between video quality and audience interaction metrics across YouTube, Bilibili, and TikTok in their analysis of laryngeal carcinoma videos. Similarly, Cheng et al. reported weak correlations between video quality scores and viewer engagement. Among the three platforms examined, TikTok videos attracted the highest levels of user interaction but demonstrated the lowest educational quality (24). Algorithm-driven prioritization of highly engaging content may therefore amplify medically insufficient videos, posing particular risks for younger users or newly diagnosed patients who may lack the health literacy necessary to critically evaluate health-related information.

These findings highlight a structural deficiency in how social media platforms manage health education content. The issue lies not only in the type of content creators but also in how platforms algorithmically rank and promote videos. Contributions from professional institutions such as transplant centers, academic hospitals, and public health organizations remain limited. This lack of representation is especially problematic in complex medical fields like organ transplantation, where accurate and comprehensive information is critical.

Improving the quality of kidney transplantation-related content on social media requires a coordinated response involving multiple stakeholders. First, physicians and transplant specialists should be supported in taking a more proactive role in public education. Prior studies have shown that clinician-generated online content improves accuracy and patient trust, particularly when supported by institutional training and structured communication frameworks (25). Medical institutions can therefore facilitate this process through formal training programs, standardized communication templates, and incentives for producing evidence-based digital content. Second, social media platforms should incorporate content-quality indicators into their recommendation systems. Systematic reviews suggest that current platform algorithms largely prioritize engagement rather than informational rigor, thereby amplifying low-quality medical content (23). Integrating reliability-based ranking signals such as expert verification, peer-review mechanisms, or credibility scoring systems could enhance the visibility of high-quality transplant education content. Third, improving public digital health literacy is essential. Recent systematic reviews demonstrated that digital-literacy interventions significantly enhance users' ability to identify trustworthy online health information, particularly in chronic-disease management and treatment-decision contexts (26, 27). Hospitals and transplant centers could incorporate digital-literacy training into routine pre- and post-transplant counseling to strengthen patients' capacity to evaluate online resources. Fourth, emerging technologies such as artificial intelligence can play a central role in improving content accuracy and personalization. Evidence shows that AI-assisted content generation improves message consistency, reduces misinformation, and can be used to guide users toward reliable, evidence-based information sources (28). AI-driven screening tools may also assist platforms in identifying misleading or harmful videos related to transplantation. Finally, national health authorities and professional societies should establish formal guidelines defining minimum standards for accuracy, completeness, transparency, and ethical compliance in digital medical content. Several international organizations have already advocated for such standards as part of broader digital- health governance frameworks, emphasizing their importance for safe online patient education (29).

## 5 Limitation

This study has several limitations. First, although validated scoring tools such as DISCERN, GQS, and PEMAT were used, the evaluation process was subjective and may have been influenced by observer bias. Second, the analysis was conducted at a single time point, and social media content as well as search algorithms are dynamic. Therefore, results may vary over time. Third, the study used only one search term, “kidney transplantation,” on each platform, which may have excluded relevant content using different terminology or layperson-friendly expressions.

## 6 Conclusion

This study is the first to systematically evaluate and compare the quality of kidney transplantation-related video content on two major social media platforms. While YouTube demonstrated relatively better performance than TikTok, neither platform provided consistently accurate or comprehensive educational materials. The widespread presence of low-quality videos, including those uploaded by healthcare professionals, raises concerns about potential misinformation and its impact on patient understanding, decision-making, and treatment adherence. To address these challenges, enhanced collaboration between healthcare professionals, academic institutions, and social media platforms is essential. Such efforts should aim to improve both the visibility and quality of evidence-based educational content related to organ transplantation in the digital space.

## Data Availability

The original contributions presented in the study are included in the article/Supplementary material, further inquiries can be directed to the corresponding authors.
