# Supplementary material for: Evaluating TikTok and YouTube as patient-education resources on kidney transplantation: a comparative analysis
Source: Front Public Health. 2026 Mar 13;14:1764270. doi: 10.3389/fpubh.2026.1764270 (PMC13021784; doi:10.3389/fpubh.2026.1764270)
Supplement: Supplementary file 1 [file Table_1.docx]

Supplementary Material

**Supplementary Table 1. Average score per DISCERN question among all included TikTok/YouTube videos**

|  | **Question** | **Average score (TikTok/YouTube)** |
| --- | --- | --- |
| Section 1 | | |
| 1 | Are the aims clear? | 3.41/3.29 |
| 2 | Does it achieve its aims? | 2.83/3.09 |
| 3 | Is it relevant? | 2.39/2.77 |
| 4 | Is it clear what sources of information were used to compile the publication (other than the author or producer)? | 1.88/2.57 |
| 5 | Is it clear when the information used or reported in the publication was produced? | 2.87/2.22 |
| 6 | Is it balanced and unbiased? | 3.61/3.81 |
| 7 | Does it provide details of additional sources of support and information? | 1.25/1.66 |
| 8 | Does it refer to areas of uncertainty? | 1.52/1.48 |
| Section 2 | | |
| 9 | Does it describe how each treatment works? | 1.40/1.73 |
| 10 | Does it describe the benefits of each treatment? | 1.52/1.77 |
| 11 | Does it describe the risks of each treatment? | 1.30/1.39 |
| 12 | Does it describe what would happen if no treatment is used? | 1.50/1.44 |
| 13 | Does it describe how the treatment choices affect overall quality of life? | 2.14/1.90 |
| 14 | Is it clear that there may be more than 1 possible treatment choice? | 1.71/1.79 |
| 15 | Does it provide support for shared decision making? | 2.10/1.84 |
| Section 3 | | |
| 16 | Based on the answers to all of these questions, rate the publication’s overall quality as a source of information about treatment choices. | 2.30/2.37 |

**Supplementary Table 2. Global Quality scale (GQS)**

|  | **Description** | **Average score (TikTok/YouTube)** |
| --- | --- | --- |
| Score 1 | Videos have poor flow, poor quality of the video, most information missing and are not at all useful for patients. | 2.05/2.47 |
| Score 2 | Videos have generally poor quality, some information listed and are of very limited use to patients. |  |
| Score 3 | Videos have moderate quality, and some important information is adequately discussed. |  |
| Score 4 | Videos have good flow, good quality, and most of the relevant information is listed and useful for patients. |  |
| Score 5 | Videos have excellent flow and quality and are very useful for patients. |  |
